# Apoptosis and Bax Expression are Increased by Coal Dust in the Polycyclic Aromatic Hydrocarbon-Exposed Lung

**DOI:** 10.1289/ehp.8906

**Published:** 2006-05-18

**Authors:** Mohamed M. Ghanem, Lori A. Battelli, Robert R. Mercer, James F. Scabilloni, Michael L. Kashon, Jane Y.C. Ma, Joginder Nath, Ann F. Hubbs

**Affiliations:** 1 Genetics and Developmental Biology Program, West Virginia University, Morgantown, West Virginia, USA; 2 Health Effect Laboratory Division, National Institute for Occupational Safety and Health, Centers for Disease Control and Prevention, Morgantown, West Virginia, USA

**Keywords:** apoptosis, Bax, caspase, coal dust, CYP1A1, CYP2B1, modifiers, polycyclic aromatic hydrocarbons, pneumoconiosis, xenobiotic metabolism

## Abstract

**Background:**

Miners inhaling respirable coal dust (CD) frequently develop coal workers’ pneumoconiosis, a dust-associated pneumoconiosis characterized by lung inflammation and variable fibrosis. Many coal miners are also exposed to polycyclic aromatic hydrocarbon (PAH) components of diesel engine exhaust and cigarette smoke, which may contribute to lung disease in these workers. Recently, apoptosis was reported to play a critical role in the development of another pneumoconiosis of miners, silicosis. In addition, CD was reported to suppress cytochrome P450 1A1 (CYP1A1) induction by PAHs.

**Methods:**

We investigated the hypothesis that apoptosis plays a critical role in lung injury and down-regulation of CYP1A1 induction in mixed exposures to CD and PAHs. We exposed rats intratracheally to 0.0, 2.5, 10.0, 20.0, or 40.0 mg/rat CD and, 11 days later, to intraperitoneal β-naphthoflavone (BNF), a PAH. In another group of rats exposed to CD and BNF, caspase activity was inhibited by injection of the pan-caspase inhibitor Q-VD-OPH [quinoline-Val-Asp (OMe)-CH_2_-OPH].

**Results:**

In rats exposed to BNF, CD exposure increased alveolar expression of the proapoptotic mediator Bax but decreased CYP1A1 induction relative to BNF exposure alone. Pan-caspase inhibition decreased CD-associated Bax expression and apoptosis but did not restore CYP1A1 activity. Further, CD-induced lung inflammation and alveolar epithelial cell hypertrophy and hyperplasia were not suppressed by caspase inhibition.

**Conclusions:**

Combined BNF and CD exposure increased Bax expression and apoptosis in the lung, but Bax and apoptosis were not the major determinants of early lung injury in this model.

Coal miners are commonly exposed to coal dust (CD) and polycyclic aromatic hydrocarbons (PAHs). The CD originates within the mine, whereas the PAHs are components of occupational and avocational exposures such as diesel engine exhaust and cigarette smoke. The CD exposure can cause coal workers’ pneumoconiosis, a disease of coal miners characterized by the aggregation of dust-laden macrophages near the respiratory bronchioles to form structures known as macules. Inflammation is a consistent feature of the pulmonary response to respirable CD (Ghanem et al. 2004; Rom et al. 1987). Although chronic inflammation is increasingly believed to play a role in carcinogenesis in many tissues (Marx 2004), the role of particle-induced inflammation in pulmonary carcinogenesis, the influence of particle-induced inflammation on the metabolism of PAHs and other known carcinogens, and the histopathologic alterations produced by combined exposure to CD and PAHs remain incompletely investigated.

PAHs are metabolized to reactive intermediates by cytochrome P450 1 (CYP1) gene products. Recently, our laboratory has demonstrated that respirable CD inhibits PAH-induced CYP1A1 activity in the lung of rats. The down-regulation of CYP1A1 induction was associated with inflammation (Ghanem et al. 2004). This information suggests that CD may modify the metabolism of PAHs in the lung. However, molecular changes that are associated with CD exposure and resulting pulmonary inflammation and down-regulation of CYP1A1 induction have not yet been identified. In an experimental model of another pneumoconiosis, silicosis, caspase inhibition with resulting apoptosis down-regulation reduces silica-induced inflammation (Borges et al. 2002). Caspases play an important role in both the intrinsic and extrinsic apoptotic pathways (Droin and Green 2004), making their inhibition an elegant tool for investigating the overall role of caspase-dependent apoptosis in the pneumoconioses (Borges et al. 2002). Because apoptosis is believed to play an essential role in the inflammation associated with one pneumoconiosis, acute silicosis, and because inflammation increased as CYP1A1 activity decreased in the rat model of coal workers’ pneumoconiosis (Ghanem et al. 2004), we investigated the hypothesis that apoptosis plays a critical role in lung injury and down-regulation of CYP1A1 induction in mixed exposures to CD and the model PAH β-naphthoflavone (BNF).

## Materials and Methods

### Animals

Male Sprague-Dawley rats [Hla(SD)CVF] were purchased from Hilltop Labs (Scottdale, PA) and kept in a barrier animal facility approved by the Association for Assessment and Accreditation of Laboratory Animal Care International. Food and water were supplied *ad libitum*. Rats were housed in ventilated shoebox cages on autoclaved hardwood (Beta-Chip; Northeastern Products Inc., Warrensburg, NY) and cellulose bedding (ALPHA-dri; Shepherd Specialty Papers, Watertown, TN) in filtered, ventilated cage racks (Thoren Caging System Inc., Hazleton, PA). Rats were acclimatized for 1 week before experiments. The animals were treated humanely and with regard for alleviation of suffering. The experimental protocol was reviewed and approved by the Institutional Animal Care and Use Committee.

### Experimental design

We conducted two separate experiments as part of this study. The first experiment was a 2-week CD dose–response experiment, in which we randomized 40 male Sprague-Dawley rats (220–270 g body weight at time of exposure) into five groups (eight rats per group) using a JavaScript random-number–generating program (Research Randomizer; Urbaniak and Plous 2003). We intratracheally (IT) instilled 0.0, 2.5, 10.0, 20.0, or 40.0 mg/rat CD suspended in sterile saline to model CD exposures of coal miners as revealed in autopsy studies (Douglas et al. 1986; Kuempel et al. 2001). Eleven days later, we intraperitoneally (IP) injected the classic CYP1A1 inducer BNF (50 mg/kg) in all rats to model the PAH-exposed lung. Two weeks after CD exposure, rats were euthanized by an overdose of sodium pentobarbital. This experiment determined the relationship between CD exposure and expression of the apoptosis mediator Bax. This study also localized Bax expression relative to the expression of CYP1A1 and cytokeratin 8/18, an alveolar type II cell marker.

The second experiment was designed to inhibit apoptosis in rats exposed to CD and BNF. In this experiment, we randomized 24 male Sprague-Dawley rats (67–93 g body weight at time of exposure) into four groups (Table 1). We instilled each rat IT with either CD (40 mg/rat) suspended in 0.3 mL saline or saline alone (controls). To inhibit apoptosis, we injected the rats IP with the caspase inhibitor Q-VD-OPH [quinoline-Val-Asp(OMe)-CH_2_-OPH; 15 mg/kg] dissolved in the vehicle [dimethylsulfoxide (DMSO)] or the vehicle alone (controls) on the day of CD exposure. We injected 10 mg/kg Q-VD-OPH on days 5, 9, 10, 11, 12, and 13 postexposure to maintain caspase inhibition, whereas controls received injections of the vehicle. Eleven days after CD exposure, we induced CYP1A1 in all rats by IP injection of BNF (50 mg/kg) in corn oil. This allowed us to determine if apoptosis was etiologically associated with lung changes seen in CD-exposed rats, including *a*) suppression of CYP1A1 activity and CYP1A1 tissue expression, *b*) suppression of CYP2B1 activity, and *c*) histopathologic alterations in lung tissue. In addition, we evaluated Bax expression and apoptosis to determine the efficacy of *in vivo* caspase inhibition with Q-VD-OPH.

### Preparation of CD suspension

CD particles from the Pittsburgh coal seam were a generous gift from V. Vallyathan (National Institute for Occupational Safety and Health). These were separated and characterized as previously described (Vallyathan et al. 1988). The particles were representative low-silica–content CD particles, with 2.3% of the particles numerically being silica. The CD particles were < 5 μm in diameter with a surface area of 7.4 m^2^/g and a mass median aerodynamic diameter of 3.4 μm. The particles contained 0.34% total iron with a 0.119% surface iron content. We sterilized the particles in an oven at 160°C for 2 hr. We freshly suspended the sterile CD particles in nonpyrogenic sterile 0.9% saline (Abbott Laboratories, North Chicago, IL) by vortexing and shaking before instillation.

### Preparation of apoptosis inhibitor Q-VD-OPH

We prepared solutions of 2% (wt/wt) Q-VD-OPH (Enzyme Systems Products Inc., Livermore, CA) in endotoxin-free DMSO (Sigma Chemical Co., St. Louis, MO).

### Preparation of BNF

We prepared a 50 mg/mL suspension of BNF (Sigma-Aldrich, St. Louis, MO) as previously described (Ghanem et al. 2004).

### Necropsy of rats

Rats were euthanized by IP injection of sodium pentobarbital (≥ 100 mg/kg) (Sleepaway; Fort Dodge Animal Health, Fort Dodge, IA) 2 weeks after CD instillation. The right mainstem bronchus was ligated, and right lung lobes were collected and immediately placed on ice for isolation of microsomes. The left lung lobe was inflated with 3.0 mL of 10% neutral buffered formalin, trimmed the same day, processed in a tissue processor overnight, and embedded in paraffin the following morning.

### Measurement of CYP1A1 and CYP2B1 protein and activity

Microsomes were prepared as previously described (Ma et al. 2002). We measured 7-ethoxyresorufin-*O*-deethylase (EROD) and 7-pentoxyresorufin-*O*-deethylase (PROD) activities as previously described (Burke et al. 1985; Ma et al. 2002). We conducted lung microsome Western blots as previously described (Ghanem et al. 2004) with minor adaptation by using a 15-well Novex tris glycine gel (Invitrogen Life Technologies, Carlsbad, CA) and 30 μg protein. Densitometry values were expressed as the percentage of the CYP1A1- or CYP2B1-positive controls.

### Bax immunofluorescence

We deparaffinized lung sections and blocked nonspecific binding as previously described (Ghanem et al. 2004). We rinsed the slides with distilled water and incubated them with a 1:20 dilution of affinity-purified polyclonal rabbit anti-Bax antibody (sc-526; Santa Cruz Biotechnology Inc., Santa Cruz, CA) overnight at room temperature followed by 2 hr at 37°C. Alexa 594-conjugated goat anti-rabbit antibody (Molecular Probes, Eugene, OR) was the secondary antibody. Nonimmune rabbit serum (BioGenex, San Ramon, CA) was used for the negative control.

### Immunofluorescence for CYP1A1 and cytokeratin 8/18

We conducted double-label immunofluorescence for localization of CYP1A1 and cytokeratin 8/18 as previously described (Ghanem et al. 2004).

### Triple-label immunofluorescence for Bax, CYP1A1, and cytokeratin 8/18

Triple-label immunofluorescence for Bax, CYP1A1, and cytokeratin 8/18 localizes the expression of Bax and CYP1A1 in alveolar type II cells (alveolar type II cells contain abundant cytokeratin 8 and cytokeratin 18). We simultaneously applied polyclonal antibodies for Bax (rabbit anti-Bax; Santa Cruz Biotechnology), CYP1A1 (goat anti-CYP1A1; Santa Cruz Biotechnology), and cytokeratin 8/18 (guinea pig anti-cytokeratin 8/18; Research Diagnostics, Flanders, NJ) at dilutions of 1:20, 1:10, and 1:5, respectively, and incubated the slides overnight in a humidified chamber at room temperature followed by 2 hr at 37°C. The slides were then incubated with a secondary antibody mixture containing Alexa 594-conjugated donkey anti-rabbit IgG (Molecular Probes), Alexa 350-conjugated donkey anti-goat IgG (Molecular Probes), and fluorescein isothiocyanate–labeled donkey anti-guinea pig IgG (Research Diagnostics) antibodies for detection of Bax, CYP1A1, and cytokeratin 8/18, respectively. When viewed with the fluorescent microscope, Bax appears red, CYP1A1 appears blue, and cytokeratin 8/18 appears green.

### Immunofluorescence digital imaging

We examined the slides with an Olympus fluorescent photomicroscope (Olympus AX70; Olympus American Inc., Lake Success, NY) using three filters: green (460–500 nm excitation), red (532.5–587.5 nm excitation) and blue (460–500 nm excitation). For each emission wavelength, we photographed five images from the proximal alveolar region, which are alveoli located near the first alveolar ducts from the terminal bronchioles, the alveolar region most affected by particle deposition. We used a Quantix digital camera (Quantix Photometrics, Tucson, AZ) with QED camera plug-in software (QED Imaging Inc., Pittsburgh, PA), and held contrast, brightness and gamma settings constant.

### Quantifying Bax immunofluorescence

We measured the area of Bax immunofluorescence in the alveolar septum using a MetaMorph Imaging System (Molecular Devices Corp., Downingtown, PA). In addition, we counted the number of cells expressing Bax per 40× field.

### Triple-label immunofluorescence morphometry

We used the MetaMorph Imaging System to measure the area of alveolar tissue labeled by fluorochromes indicating expression of Bax (red), CYP1A1 (blue), and cytokeratin 8/18 (green). We also measured the co-localization of Bax, CYP1A1, and cytokeratin 8/18. Proportional CYP1A1 expression in alveolar type II cells corrected for particle-induced increases in the cells and was the area of blue fluorescence (representing CYP1A1) co-localized with green (representing cytokeratin 8/18) divided by the total green area. In each 40× microscopic field, we also counted the number of alveolar type II cells expressing Bax (red cells) and the number of alveolar type II cells expressing both CYP1A1 and Bax.

### TUNEL assay

We conducted terminal deoxynucleotidyl transferase-mediated dUTP nick end-labeling (TUNEL) as previously described (Gavrieli et al. 1992) using a TUNEL assay kit (Promega, Madison, WI). Briefly, we incubated deparaffinized slides with the terminal deoxynucleotidyl transferase enzyme and the nucleotide mix. We applied propidium iodide as a counterstain. For a positive control, we incubated slides with DNase 1 (Sigma-Aldrich) and for a negative control, we incubated slides without the terminal deoxynucleotidyl transferase enzyme.

### Histopathology

A board-certified veterinary pathologist (A.F.H.) evaluated the lung sections while blinded to the exposure status. Histopathologic changes were semiquantitatively scored on a scale ranging from 0 to 5 for both severity and distribution to produce a sum pathology score of 0 to 10 for each slide (Hubbs et al. 1997).

### Statistics

We analyzed data using SAS/STAT software (version 8.2 of SAS System for Windows; SAS Institute Inc., Cary, NC). For Bax area and Bax-labeled cells per field, we used the Proc Reg procedure. We analyzed variables in the CD response study with a one-factor analysis of variance (ANOVA) using the Proc Mixed procedure. We analyzed effects of the caspase inhibitor using a two-factor ANOVA and compared different alveolar regions using a three-factor split-unit ANOVA using Proc Mixed. We used Fisher’s least significant difference test for all post hoc pairwise comparisons. We used the nonparametric Kruskal-Wallis test followed by the Wilcoxon rank-sum test for pairwise comparisons of pathology scores. All results were considered statistically significant at *p* ≤ 0.05.

## Results

### Effect of CD exposures on alveolar Bax expression

**Figure 1:**
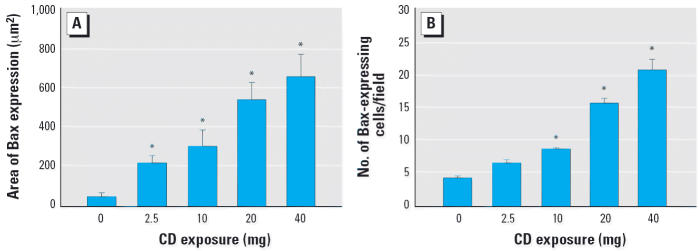
Quantification (mean ± SE) of Bax expression in the proximal alveolar region with increasing CD exposure in BNF-exposed rats. (*A*) Area of Bax expression. (*B*) The number of cells positive for Bax. *Significantly different from saline/BNF (*p* ≤ 0.05).

In the proximal alveolar region, CD increased the area of Bax expression in a dose-dependent manner (*r*^2^ = 0.6541, *p* ≤ 0.001) (Figure 1A; Supplemental Figure 1; available online at http://www.ehponline.org/docs/2006/8906/suppl.pdf). In addition, the average number of lung cells expressing Bax in the proximal alveolar region was increased in a dose-dependent fashion by CD exposure (*r*^2^ = 0.903, *p* ≤ 0.001) (Figure 1B; Supplemental Material, Figure 1, available online at http://www.ehponline.org/docs/2006/8906/suppl.pdf).

### Relationship between Bax expression and CYP1A1 induction in CD-exposed rats

**Figure 2:**
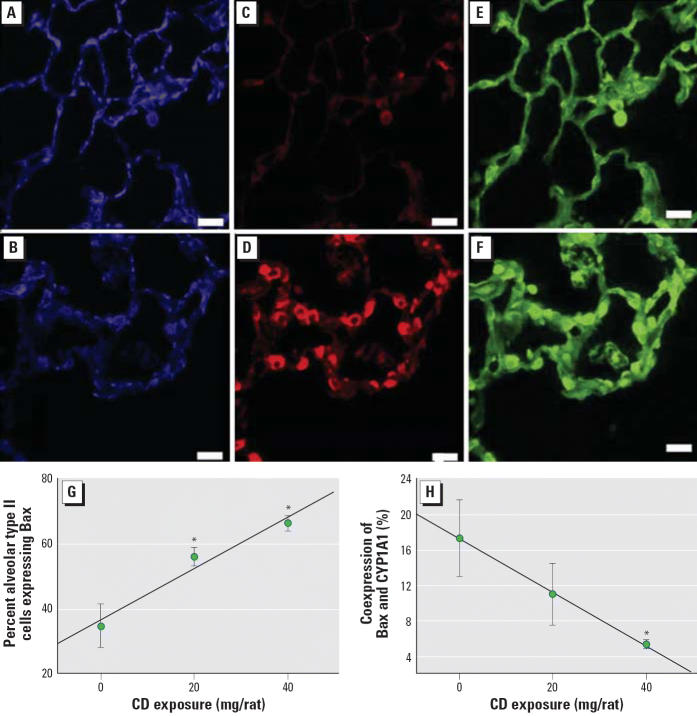
Triple-label immunofluorescence for CYP1A1, Bax, and cytokeratin 8/18 in the BNF-exposed pulmonary alveolus in response to CD exposure. (*A*) CYP1A1 expression in a BNF-exposed rat. (*B*) CYP1A1 expression in a rat exposed to CD and BNF. (*C*) Bax expression in a BNF-exposed rat. (*D*) Increased Bax expression in a CD- and BNF-exposed rat. (*E*) Expression of cytokeratin 8/18 (alveolar type II cell marker) in a BNF-exposed rat. (*F*) Increased expression of cytokeratin 8/18 in a BNF- and CD-exposed rat. Blue fluorescence indicates CYP1A1, red indicates Bax, and green indicates cytokeratin 8/18; bars = 20 μm. (*G*) The percentage of alveolar type II cells expressing Bax is increased by CD exposure. (*H*) The percentage of alveolar type II cells expressing both Bax and CYP1A1 is decreased by CD exposure. Values shown are mean ± SE. *Significantly different from saline/BNF (*p* ≤ 0.05).

In rats exposed to BNF, CYP1A1 was strongly expressed (Figure 2A); however, CD exposure decreased BNF-induced CYP1A1 expression (Figure 2B). In rats exposed to BNF alone, Bax was rarely expressed (Figure 2C), but it was frequently expressed in alveolar cells from rats exposed to CD and BNF (Figure 2D). Small numbers of cytokeratin 8/18–containing alveolar type II cells were observed in rats exposed to BNF alone (Figure 2E). CD increased the size and number of alveolar type II cells in BNF-exposed rats (Figure 2F).

The percentage of alveolar type II cells coexpressing Bax was marginally increased (*p* = 0.052) with CD exposure (Figure 2G; Supplemental Material, Figure 2; available online at http://www.ehponline.org/docs/2006/8906/suppl.pdf). The percentage of alveolar type II cells that concomitantly expressed both Bax and CYP1A1 was significantly decreased (*p* = 0.02) with increasing CD exposure (Figure 2H; Supplemental Material, Figure 2; available online at http://www.ehponline.org/docs/2006/8906/suppl.pdf), suggesting an inverse relationship between CYP1A1 induction and Bax expression in alveolar type II cells.

### Effect of caspase inhibition on proximal alveolar region Bax expression in CD-exposed rats

**Figure 3:**
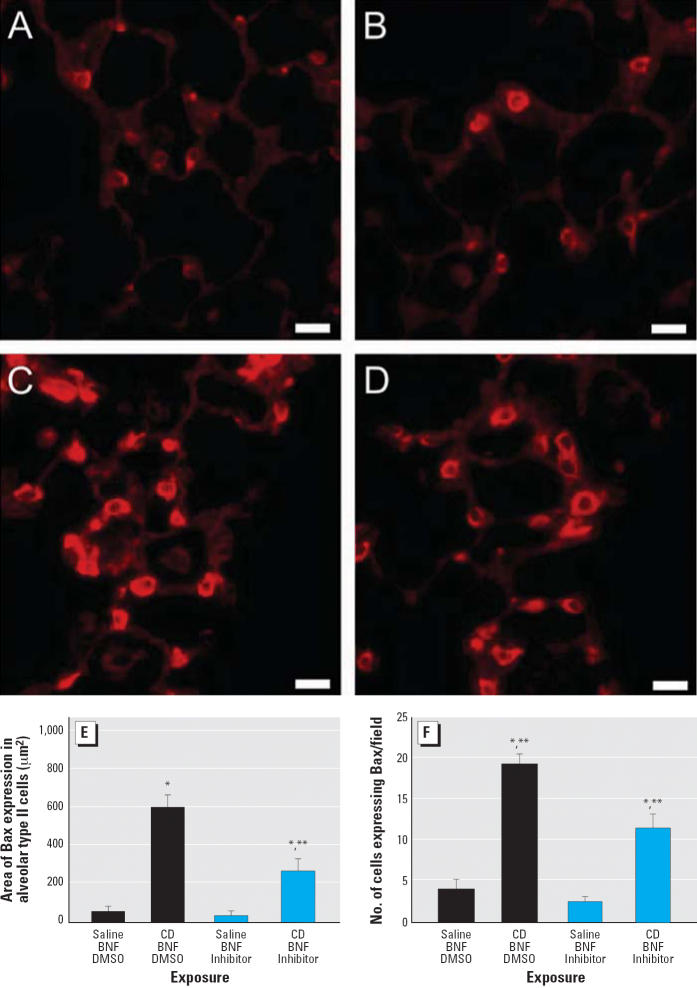
Immunofluorescent staining showing suppression of Bax expression by Q-VD-OPH in the proximal alveolar region of rats receiving (*A*) saline/BNF/DMSO, (*B*) saline/BNF/caspase inhibitor, (*C*) CD/BNF/DMSO, or (*D*) CD/BNF/caspase inhibitor. Bars = 20 μm. (*E*) Bax expression area localized to alveolar type II cells. (*F*) The number of Bax-expressing cells. Values shown are mean ± SE. *Significantly different from saline/BNF (*p* ≤ 0.05). **Significantly different from corresponding rats not receiving Q-VD-OPH.

In rats exposed to both CD and BNF, Q-VD-OPH significantly reduced the area of Bax expression (*p* < 0.001) (Figure 3A–E) and the number of cells expressing Bax per field (*p* = 0.001) (Figure 3A–D,F). However, in BNF-exposed rats, Q-VD-OPH did not completely eliminate CD-associated increases in Bax expression (Figure 3D–F); the area of Bax expression (Figure 3E) and the number of cells expressing Bax per field (Figure 3F) were significantly increased by CD exposure (*p* = 0.005 and *p* ≤ 0.001, respectively) despite Q-VD-OPH treatment.

### Effect of CD on induced CYP1A1 immunofluorescence: role of caspases

**Figure 4:**
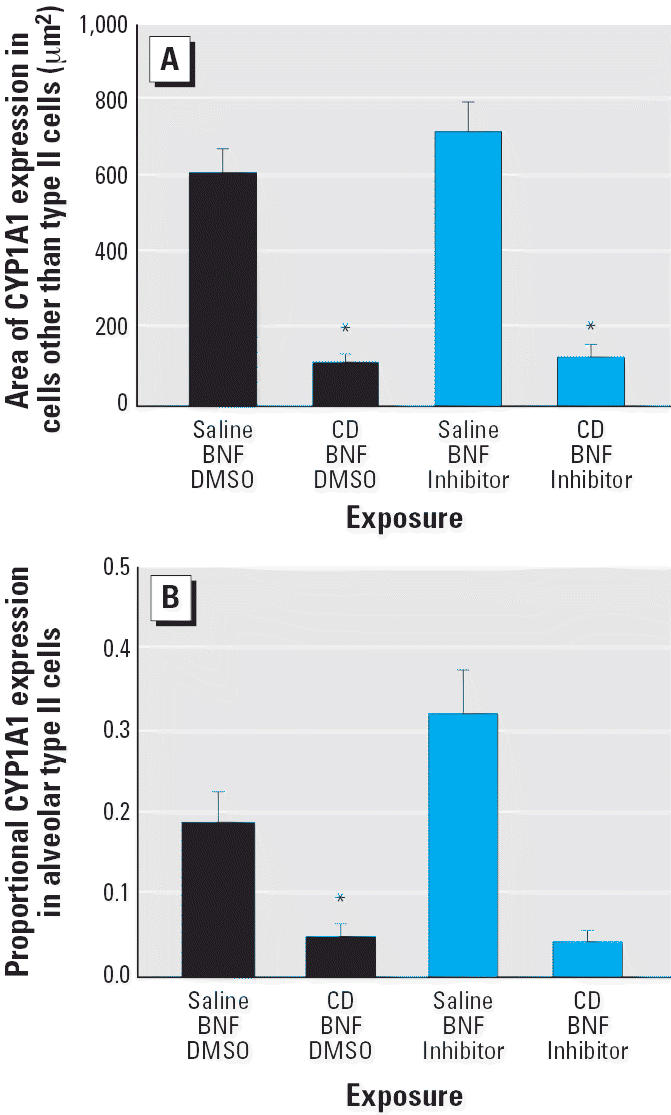
CYP1A1 immunofluorescence (mean ± SE) in the alveolar region of BNF-induced rats exposed to CD with and without pan-caspase inhibition by Q-VD-OPH. (*A*) CYP1A1 expression in alveolar cells that are not type II cells. (*B*) Proportional CYP1A1 expression area in alveolar type II cells. *Significantly different from saline/BNF (*p* ≤ 0.05).

We evaluated the effect of CD on PAH-induced CYP1A1 within alveoli in rats exposed to BNF (the model PAH) in the alveolar region of the lung most affected by particle deposition, the proximal alveolar region. Within alveolar septa stained for both CYP1A1 and cytokeratin 8/18, the area of BNF-induced CYP1A1 expression localized to cells without the alveolar type II markers (cytokeratin 8/18) was significantly reduced (*p* ≤ 0.001) by CD exposure (Figure 4A; Supplemental Material, Figure 3; available online at http://www.ehponline.org/docs/2006/8906/suppl.pdf). In addition, the proportional CYP1A1 expression in alveolar type II cells was significantly reduced (*p* = 0.0028) by CD exposure in BNF-injected rats (Figure 4B; Supplemental Figure 3, available online at http://www.ehponline.org/docs/2006/8906/suppl.pdf). Q-VD-OPH did not significantly affect CYP1A1 immunofluorescence in the alveolus of rats exposed to BNF or to BNF and CD (Figure 4).

### Effect of caspase inhibition on CD-induced apoptosis and morphologic alterations in BNF-exposed rats

**Figure 5:**
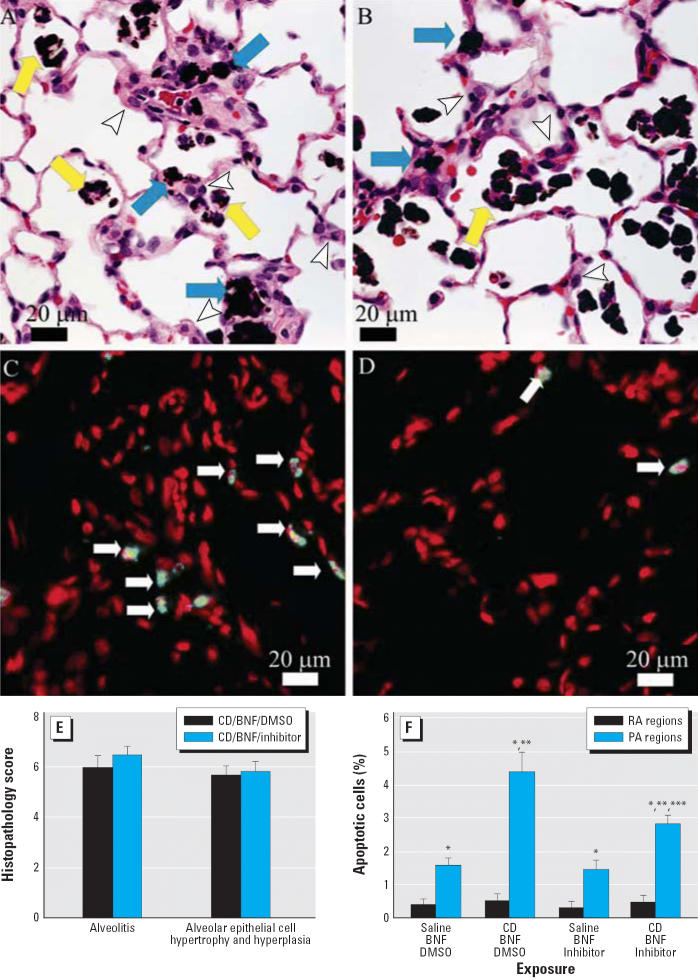
Morphologic features of caspase inhibition in CD-exposed pulmonary alveolus. (*A,B*) Lung photomicrographs demonstrating dust-laden macrophages in alveolar spaces (yellow arrows) and in the interstitium (blue arrows), with hypertrophy and hyperplasia of alveolar type II cells (arrowheads) from (*A*) a CD-exposed rat (CD/BNF/DMSO) and (*B*) a CD-exposed rat following caspase inhibition (CD/BNF/inhibitor). (*C,D*) TUNEL assay showing green fluorescent apoptotic cells (arrows) in (*C*) a CD-exposed rat (CD/BNF/DMSO) and (*D*) a CD-exposed rat after caspase inhibition (CD/BNF/inhibitor); note the decreased number of apoptotic cells compared with (*C*). Bars = 20 μm. (*E*) Semiquantitative histopathology scores with no evidence that caspase inhibition modifies CD-induced alveolitis or alveolar epithelial cell hypertrophy and hyperplasia. (*F*) Apoptosis in BNF-treated rats exposed to IT CD with and without caspase inhibition. Apoptosis is significantly higher in the proximal alveolar (PA) region when compared with random alveolar (RA) regions. CD significantly increased apoptosis; caspase inhibition significantly decreased, but did not entirely abrogate, CD-induced apoptosis. Values shown are mean ± SE. *Significantly different from random alveolar region (*p* ≤ 0.05). **Significantly different from corresponding rats not receiving CD. ***Significantly different from corresponding rats not receiving Q-VD-OPH.

Histopathologic alterations associated with CD exposure in BNF-treated rats included histiocytic and suppurative alveolitis with accumulation of dark brown particles within the cytoplasm of many alveolar macrophages, and alveolar epithelial cell hypertrophy and hyperplasia (Figure 5A). Caspase inhibition did not significantly affect these histopathologic changes of CD- and BNF-exposed rats (Figure 5B,E). We did not observe histopathologic changes in the lungs of rats instilled IT with vehicle control and injected with BNF or BNF and Q-VD-OPH. However, CD exposure significantly increased the percentage of apoptotic cells (*p* ≤ 0.048; Figure 5C) in the proximal alveolar region, whereas caspase inhibition with Q-VD-OPH significantly decreased the percentage of apoptotic cells in the proximal alveolar region of the CD-exposed rats (*p* = 0.013; Figure 5D,F). The proximal alveolar region had a higher percentage of apoptotic cells than did random alveolar regions from rats with the same exposures (*p* = 0.013, *p* ≤ 0.001, *p* = 0.025, and *p* < 0.001 for saline/BNF/DMSO, CD/BNF/DMSO, saline/BNF/inhibitor, and CD/BNF/inhibitor, respectively; Figure 5F).

### Effect of caspase inhibition on EROD and PROD

**Figure 6:**
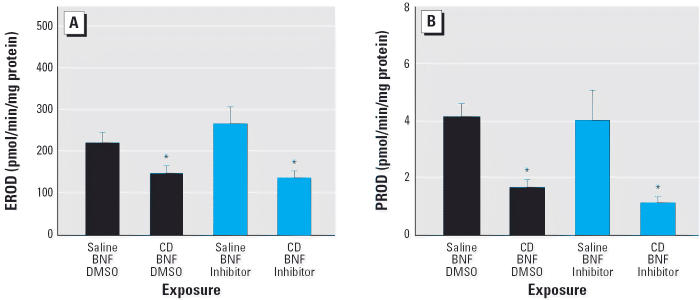
CD significantly decreases both CYP1A1- and CYP2B1-dependent metabolism in the lung, shown by EROD activity (*A*) and PROD activity (*B*) of the BNF-exposed lung. Values shown are mean ± SE. *Significantly different from rats not exposed to CD (*p* ≤ 0.05).

Two forms of CYP were selected for evaluation, the PAH-inducible CYP1A1 and the major constitutive isoform of the rat lung, CYP2B1. To determine if Bax expression or apoptosis inhibited the activity of CYP1A1- or CYP2B1-dependent reactions, we measured pulmonary EROD and PROD activity. BNF-induced EROD activity was significantly reduced (*p* ≤ 0.05) in CD-exposed rats but unaffected by the pan-caspase inhibitor Q-VD-OPH (Figure 6A). Also, CD significantly reduced PROD activity in BNF-treated rats, and this effect persisted when caspases were inhibited by Q-VD-OPH (Figure 6B).

### Effect of caspase inhibition on CYP1A1 and CYP2B1 Western blots

**Figure 7:**
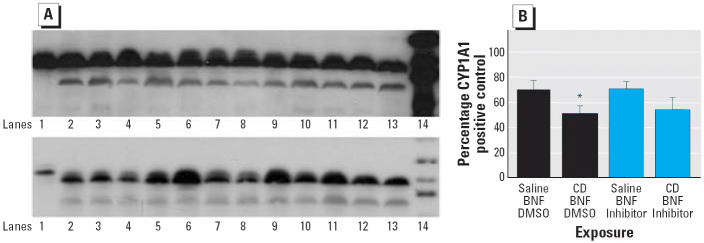
Effect of CD on lung CYP1A1 Western blots. (*A*) Representative Western blots of lung microsomes. In the top panel, lane 1 is the CYP1A1 control, lanes 2–7 are from BNF-exposed rats, lanes 8–13 are from BNF- and CD-exposed rats, and lane 14 is the molecular weight marker. In the bottom panel, lane 1 is the CYP1A1 control, lanes 2–6 are from rats exposed to BNF and caspase inhibitor; lanes 7–12 are from rats exposed to BNF, CD, and caspase inhibitor; lane 13 is from a rat exposed to both CD and BNF, and lane 14 is the molecular weight marker. (*B*) Densitometry values (mean ± SE). *Significantly different from rats not exposed to CD (*p* ≤ 0.05).

CD exposure significantly reduced the amount of CYP1A1 protein in the lungs of BNF-exposed rats (*p* ≤ 0.05; Figure 7), but the amount of CYP1A1 protein was unaffected by caspase inhibition with Q-VD-OPH (Figure 7). Similarly, the amount of CYP2B1 was not significantly affected by caspase inhibition with Q-VD-OPH (data not shown).

## Discussion

High tissue concentrations of respirable CD accumulate in the human lung in coal miners. For example, in an autopsy study, the actual measured dust burden (mean ± SD) in the lung of nonsmoking coal miners was 16.4 ± 8.5 g (Kuempel et al. 2001). However, most epidemiologic studies indicate no increased risk of lung cancer in coal miners (Kuempel et al. 1995). A potential difficulty in interpreting the human lung cancer epidemiology studies of CD is the potential confounding from exposure to tobacco, a known lung carcinogen that contains abundant PAHs. This is extremely important in coal miners because in studies where smoking status is known, most coal miners are smokers (Vallyathan et al. 1985). In addition, diesel engine exhaust and other sources of PAHs are present within coal mines. In our study, we have confirmed our previous finding that in mixed exposures to CD and PAHs, CD appears to modify PAH metabolism by inhibiting CYP1A1 induction (Ghanem et al. 2004). In the present study, we also found that CD, like many other agents that cause chronic pulmonary inflammation and fibrosis, increases pulmonary apoptosis and increases expression of the pro-apoptotic mediator Bax. Previous studies suggest that apoptosis plays a role in the pathogenesis of a diverse group of fibrosing lung diseases, including diffuse alveolar damage, idiopathic pulmonary fibrosis (Kuwano et al. 1999), and silicosis (Borges et al. 2002).

In this study, we demonstrate for the first time that *in vivo* treatment with the pan-caspase inhibitor Q-VD-OPH significantly decreases apoptosis and Bax expression in the lung. Because caspases are mediators of apoptosis, decreased apoptosis is an expected result of caspase inhibition (Caserta et al. 2003). Although somewhat unexpected, caspase inhibition of Bax expression in our study suggests that caspases play a role in Bax expression in this model. Importantly, immunohistochemical detection of Bax expression in lung correlates well with apoptosis and the expression of other apoptosis mediators (Kuwano et al. 2000; Plataki et al. 2005). However, the relationship between increased Bax expression in tissue sections and Bax activation has not been investigated thoroughly.

CD-associated histopathologic alterations, such as alveolitis and alveolar epithelial cell hypertrophy and hyperplasia, were unaffected by caspase inhibition and decreased apoptosis. Other pan-caspase inhibitors, such as *N*-benzyloxy-carbonyl-Val-Ala-Asp-(*O*-methyl)-fluoromethyl ketone and BOC-Asp(*O*-methyl)-fluoromethyl ketone, have been reported to reduce neutrophil accumulation in the lungs of silicotic mice by 50% (Borges et al. 2002). However, in the present study, the principally histiocytic inflammatory reaction in the lungs of CD-exposed rats was not significantly suppressed by the injection of Q-VD-OPH. Because the histopathologic changes associated with silica exposure involve more neutrophils and are more proliferative than CD-induced pulmonary injury (Green 2000; Kuempel et al. 2003), these morphologic differences may influence the ability to attenuate silicotic injury through apoptosis inhibition. It may also be that the role of apoptosis in CD- and PAH-induced lung injury is similar to the role of apoptosis in oxygen toxicity, where apoptosis contributes to cell death but caspase inhibition does not significantly alter alveolar damage (Barazzone et al. 1998). Alternatively, Bax expression and apoptosis in the CD- and PAH-exposed lung may be involved in resolution of lung injury, as has been suggested in the lipopolysaccharide-exposed lung (Wang et al. 2002).

The present study clearly indicates that apoptosis is not a major contributor to early morphologic injury in the CD- and PAH-exposed rat lung. Changes in cellular mediators or signaling cascades that produce Bax activation and increase apoptosis may have a role in CYP down-regulation. Potential candidates would include mediators of particle-induced inflammation including p53, nitric oxide, nuclear factor-κB (NF-κB), and/or initiators of alveolar epithelial proliferation. Indeed, *in vitro* studies indicate down-regulation of CYP1A1 induction by tumor necrosis factor α and activated NF-κB (Tian et al. 2003).

In summary, our findings indicate that, in mixed exposures to CD and PAHs, CD modifies important processes associated with the pulmonary response to xenobiotics, notably down-regulating xenobiotic metabolism and increasing apoptosis. However, our findings do not support the hypothesis that apoptosis plays a critical role in early lung injury and the down-regulation of CYP1A1 induction in mixed exposures to CD and PAHs. Therefore, suppression of CYP2B1 and inducible CYP1A1 following CD and PAH exposure is associated with, but not caused by, up-regulation of Bax expression and apoptosis of alveolar cells.

## Figures and Tables

**Table 1:** The treatment groups, group size, and type of treatment in the caspase inhibitor study.

Group	No. of rats	IT exposure	Caspase inhibitor (IP)	PAH (IP)
1	6	Saline	DMSO	BNF
2	5	Saline	Q-VD-OPH	BNF
3	7	CD	DMSO	BNF
4	6	CD	Q-VD-OPH	BNF
