# Supplementary material for: Apoptosis and Bax Expression are Increased by Coal Dust in the Polycyclic Aromatic Hydrocarbon-Exposed Lung
Source: Environ Health Perspect. 2006 May 18;114(9):1367–73. doi: 10.1289/ehp.8906 (PMC1570065; doi:10.1289/ehp.8906)
Supplement: Supplemental Figures and Tables [file ehp0114-001367s1.pdf]

Supplementary Figure 1. Representative images of immunofluorescent single staining of Bax in rat lung showing a dose-dependent increase in the number of cells expressing Bax by CD exposure. All rats received intraperitoneal BNF to model the PAH exposed lung while the intratracheal exposure to CD varied in these representative photomicrographs. (A) Bax expression in the lung of a rat receiving no CD. (B) Bax expression in the lung of a rat exposed to 2.5 mg CD. (C) Bax expression in the lung of a rat exposed to 10 mg CD. (D) Bax expression in the lung of a rat exposed to 20 mg CD (E) Bax expression in the lung of a rat exposed to 40 mg CD. Reference bar is 20 micrometers.

Supplementary Figure 2. Photomicrographs demonstrating details of triple label immunofluorescence (IF) showing the effect of 40 mg/kg CD on CYP1A1 and Bax expression in alveolar type-II cells in the PAH exposed lung. Alveolar type-II cells are identified by expression of cytokeratins 8/18 in the alveolus. All rats were intraperitoneally exposed to the PAH, BNF, to induce CYP1A1. For morphometric assessment, the color range representing positive fluorescence was selected and area of fluorescence for CYP 1A1, Bax, and cytokeratins 8/18 and their co-localization were measured. (A) Sites of blue fluorescence labeling of CYP1A1 are indicated by the light (fluorescent) blue color in the lung of a rat receiving no CD, (B) Blue fluorescence labeling of CYP1A1 in the lung of a CD-exposed rat (C) Dual IF for Bax (red) and CYP1A1 (blue) in the same section shown in B. Co-expression of Bax and CYP1A1 results in a purple color. Bax is principally expressed in sites that do not express CYP1A1. (D) In the same area shown in A, but viewed with a red filter, occasional red fluorescence indicates the presence of rare cells expressing Bax in a rat receiving no CD.

(E) In the same area shown in B, but viewed with a red filter, increased numbers of red fluorescent cells indicates the presence of increased numbers of cells expressing Bax in a CD-exposed rat. (F) Dual IF for Bax (red) and cytokeratins 8/18 (green) in the same section from a CD-exposed rat shown in B and E. Co-expression of both Bax and cytokeratins 8/18 results in a yellow to orange color and indicates Bax expression in alveolar type II cells. Bax expression is frequently localized to alveolar type II cells, but not all alveolar type II cells express Bax. (G) In the same area shown in A and D, but viewed with a green filter, green fluorescence indicates the presence of alveolar type II cells expressing cytokeratins 8/18 in a rat receiving no CD. (H) In the same area shown in B and E, but viewed with a green filter, increased numbers of green fluorescent cells indicates increased numbers of alveolar type II cells expressing cytokeratins 8/18 in a CD-exposed rat. (I) Dual IF for CYP1A1 (blue) and cytokeratins 8/18 (green) of the same section shown in B and D showing that CYP1A1 is principally expressed in septal area that are not alveolar type II cells with very little blue-green fluorescence indicating expression of CYP1A1 in alveolar type II cells. (J) Simultaneous examination of blue, green and red fluorescence after triple labeling in the control (BNF alone) section shown in A, D, and G. (K) Simultaneous examination of blue, green and red fluorescence after triple labeling in the a section from a CD-exposed (CD exposure in a BNF treated rat). This is the same section shown in B, C, E, F, G, H, and I but viewed simultaneously with all three filters. Bar is 20 microns.

Supplementary Figure 3. Double label Immunofluorescence images for CYP1A1 and cytokeratins 8/18 in the alveolar region of the PAH-exposed rat lung. In this double labeled section of lung, CYP1A1 protein was indicated by red fluorescence, cytokeratins 8/18 were indicated by green fluorescence, and co-expression of CYP1A1 and cytokeratins 8/18 resulted in both red and green expression in the same pixels causing a yellow color (A) Photomicrograph of immunofluorescence for CYP1A1 and cytorkeratins 8/18 in the lung of a control PAH-exposed rat lung (saline/BNF/DMSO) (B) CD exposure reduced CYP1A1 (red) fluorescence while the number of cells expressing cytokeratins 8/18 (green) increased by CD in the lung in this CD/BNF/DMSO exposed rat compared to the control (saline/BNF/DMSO) rats illustrated in A. Note the distinction between the dark green autofluorescence of formalin-fixed tissue and the specific bright green fluorescence of the alveolar type II cells which express cytokeratins 8/18. (C) Photomicrograph from a PAH-exposed rat receiving the pan-caspase inhibitor, Q-VD-OPH (saline/BNF/inhibitor group). Q-VD-OPH did not significantly alter expression of CYP1A1 or cytokeratins 8/18 in the alveolar region. The response was very similar to that of the control lungs illustrated in A. (D) CYP1A1 expression was decreased in the entire alveolar septum and was proportionally reduced in AT-II cells of CD/BNF/inhibitor group illustrated in D compared to control (saline/BNF/inhibitor) group illustrated in C. AT-II hyperplasia and hypertrophy (indicated by the green color of cytokeratins 8/18) is present in both CD/BNF/inhibitor group illustrated in D and in the CD/BNF/DMSO group illustrated in B, and absent in saline/BNF/DMSO and saline/BNF/inhibitor. Reference bar is 20 micrometers.

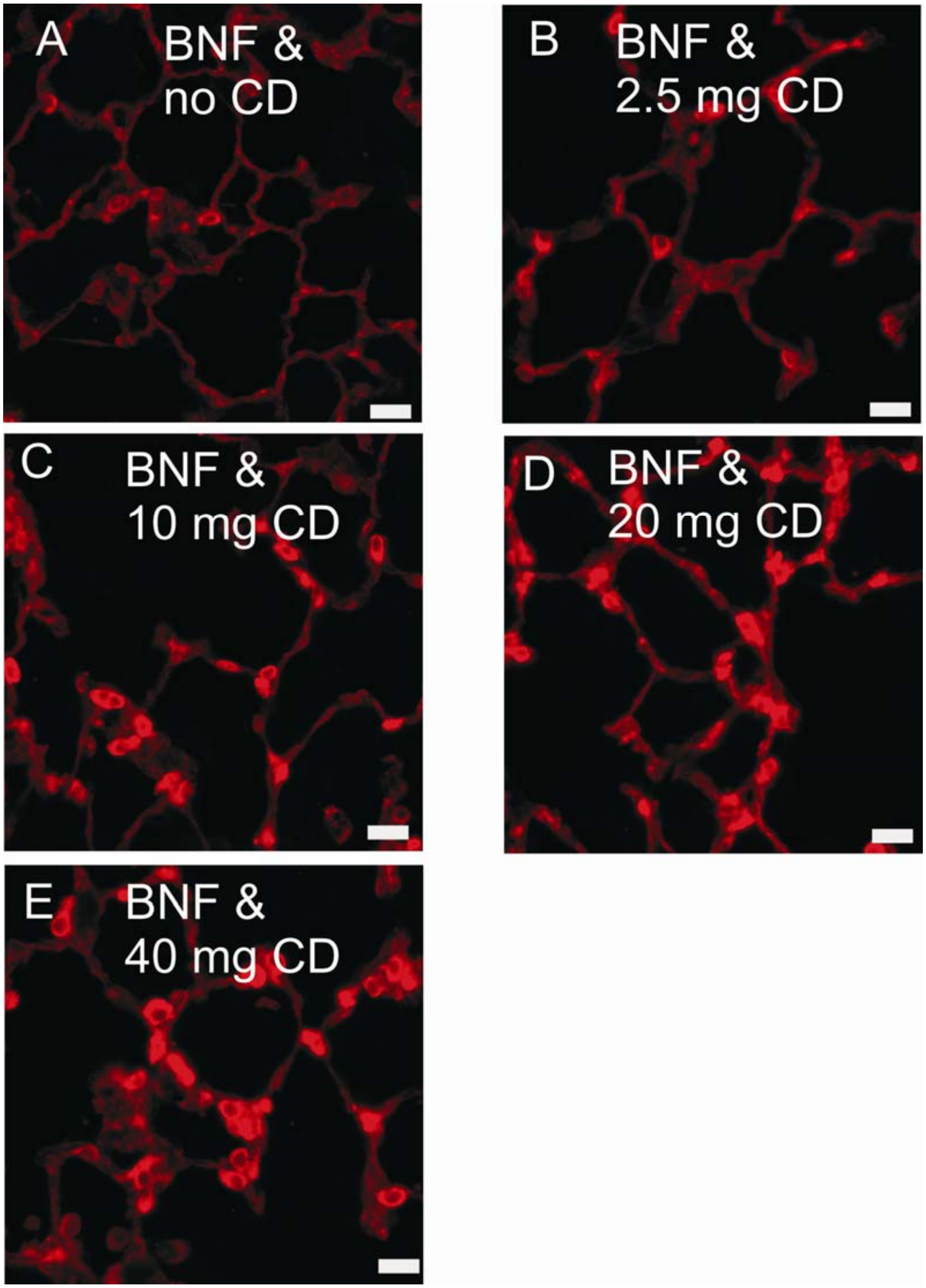

Supplementary Figure 1

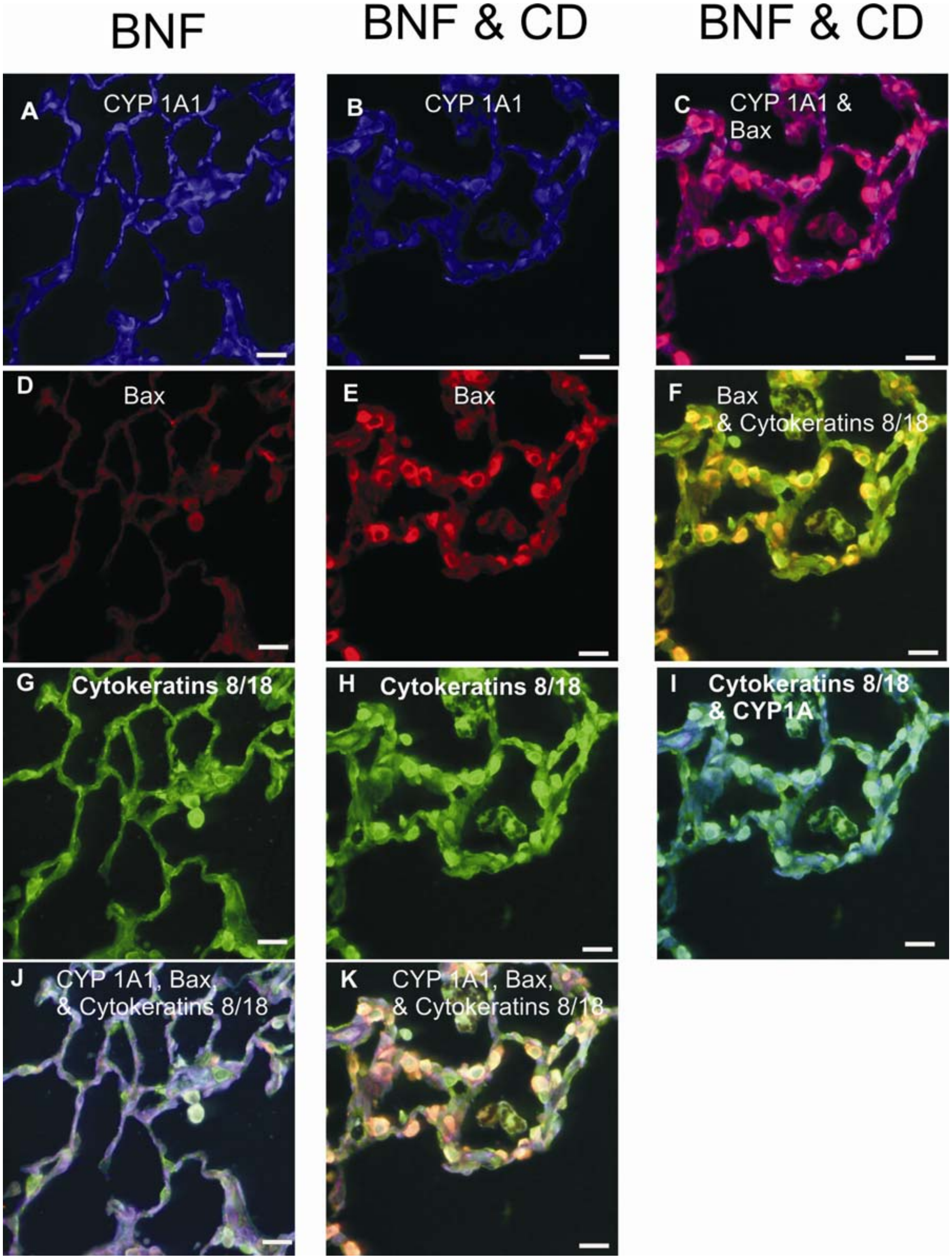

Supplementary Figure 2

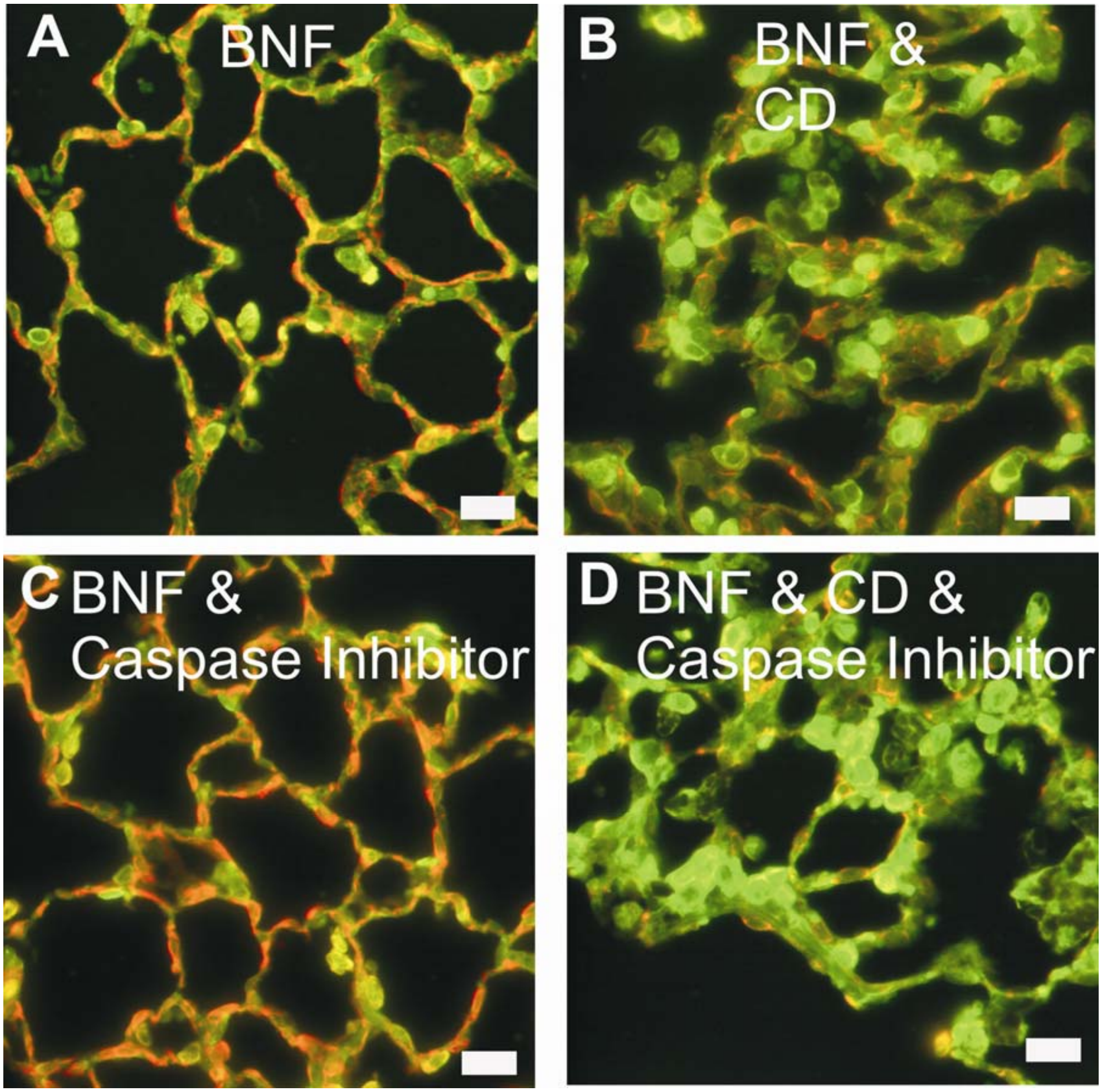

Supplementary Figure 3
